# Principles for designing accountability and responsibility plan in medical education that spreads across multiple sites

**DOI:** 10.15694/mep.2019.000057.1

**Published:** 2019-03-15

**Authors:** Lee HangFu, Samal Nauhria

**Affiliations:** 1 Windsor University School of Medicine

**Keywords:** Medical Education, Health Policy, Organizational Policy, Medical Faculty

## Abstract

This article was migrated. The article was marked as recommended.

In the era of higher competition, less clinical clerkship sites and a tighter budget, many off-shore and on-shore medical schools resort to multiple training sites to meet the needs of GMC requirements. The greatest hurdle is to maintain comparable training sites while providing the reliability and validity standards of the accreditation bodies. The lack of direct supervision and the dilution of visible accountability despite any existing organizational hierarchy structure has minimized the effectiveness of the educational program. In this paper, I have simplified and refocused the chain of command by identifying the pillars of a medical school; reiterate the importance of organizational hierarchy, communication between the pillars and within the departments, recharge the accountability directives, motivate self improvement, on-going research and individual responsibilities within and between each of the multiple sites achieving competitiveness and comparable One-University.

## Introduction

The ultimate intent of the Medical Education Program is to prepare students entering the residency matching programs and succeed in their choice in a medical career. It is the responsibility of all faculties to bring forth the Mission and Vision of the school and be accountable for the position that they are bestowed to train our future physicians.

The defining “Chain of Commands” of the Accountability and Responsibility Policies are often weakened as the chain crosses multiple sites from Pre-Clinical to Clinical and even within Multiple Clinical sites. Curricular courses are often subjected to “Shared Governance”, a misunderstood concept and junior faculties are often included in the sharing with no supervision to ensure the standardization, reliability and validity of the course contents. The Accountability and Responsibility in the Chain of Command must be identified at every level of faculty members and administrators between multiple sites with clear instructions and bearing potential consequences.

In a pursuit for the goal of excellence, the Medical Curriculum is a challenging blend of traditional and innovative programs that must be in-line with the Accreditation Committee of Medical Learning Objectives (Purposes and Functions of the Curriculum Committee, 2013). The continuity of these principles is the foundation for a successful Education Methodology that will produce the most consistent, respectable, knowledgeable, and competent graduating students achieving the ultimate career goal. This path of success is a guided journey that must be a collaborative effort from the administrative and faculties throughout the entire duration of the student’s education experience. Any deviation from the curricular program due to the lack of accountability and responsibility by any member of the facilitators with a weakened or broken chain of leadership will cause irrevocable damage to the path of success.

The Twelve Principles will assist the reader in the development of an “Accountability and Responsibility Directives”, which should focus on the institution pillars within the Medical School.

## Twelve Principles

### Principle #1: Identify Key Factors in Accountability

Accountability key factors can be grouped into four categories of teaching, leadership, curriculum, and Student involvement ( Reeves, 2004).

The effective application of accountability in material content, interaction and method of delivery will identify ordinary teachers doing extraordinary things. All educating administrator or faculties are leaders who appreciate the challenge to be accountable. They discover various aspects of their responsibility to Assessments, Chain of Supervision, and Learning Objectives that are all important contributing events in the development of the curricular program. Many school curriculum committees have engaged in curriculum Inventory (mapping), and virtually all medical schools have attempted to make certain that its curriculum is aligned with applicable accreditation standards. The ultimate educational outcome does not demonstrate a single link to accountability unless the Medical Curriculum Committee is willing to map the relationship of those curriculum Learning Objectives to actual implementation in the classroom.

The student and tutor participation in education and formative assessment clearly has a major effect on student education success. An early identification of a “watch list” of students in danger of failure can be remediated using a team approach to monitor and improve student performance. Accountability offers a better outcome for weak students when multiple channels are communicated early and tutor or student group assistance are encouraged and arranged early in their career path.

### Principle #2: Identifying the top link in the Chain of Accountability (eg: Medical Education Department - MED)

The characteristic organization of the MED should include categories of the office, division, centre and department. When appointing a leader for the department one must consider M.D. qualified, holding a tenured path possesses a master’s in medical education and can assimilate the role of the Department Chair. In order to maintain accountability of any faculty member, yearly or bi-yearly evaluation is an essential part of the tenure process. The focus of evaluation is on educational quality improvement and is equivalent to Professional Education Audit. Medical schools equate evaluation as quality assurance procedures. A purposeful evaluation is much greater than the terms of simple audit. It provides confirmation on how successful students’ learning objectives are being accomplished and teaching standards are satisfactorily delivered and enabling the curriculum to evolve ( Al Shawwa, 2012). A medical curriculum should constantly evolve in response to the needs of students, institutions, and society.

### Principle #3: Identify the Institution Pillars

#### Pillar #1: Supporting Curriculum Development (eg. Medical Curriculum Committee)

Curriculum development is one of the fundamental roots to any educational program. The processes of curriculum development require the awareness to at least five major fundamentals namely: Objective goals; Strategic outcome of intent; the activities of teaching and learning; methods of assessment; and processes for Psychometric Analysis.

Each medical school must have policy guidelines directing through the curriculum committees, on the details of the curriculum and its pedagogy objectives independently. The spectrum of educational pedagogy strategies are typically delivered as a lecture-based/teacher centered to problem-based/student-centered approach or mixed.

#### Pillar #2: Investing in Basic Science Faculty and Clinical Affiliate development (eg. Faculty Development Departments, FDD)

One of the most essential roles of the FDD is educating, improving and enriching faculty performance and develops accountability and responsibility within an institution focuses on teaching and learning. In addition, it must play the leading role of advising in the evolution of the curriculum in accordance with Best Evidence Medical Education. It leads expertise in student assessment and curriculum evaluation. FDD must be available in offering support to the development of faculty-focused instructional materials and student study resources for online and offline learning.

FDD must accept and understand that a lot of its clinical faculty and preceptors will not be able to participate in the faculty development workshop programs. The Clinical Workshops should match clinicians’ ambition, high standards, interactive and focus on specific problems facing the clinical faculty members. An open channel between FDD and Clinical Departments will assist the faculty members of the clinical department to identify their own challenges so a customized workshop will be developed to suit the needs of the Clinical Department. A monthly/quarterly FDD periodical should focus on latest issues related to recent trends, innovations, improvements, updates and changes in medical education.

#### Pillar #3: Fostering Research Process Role

It is beneficial with utmost priority to create a culture of research in the Medical School. The approach and expectations of research in medical education should not be identical to scientific research. Research in medical education should focus either the qualitative or quantitative practice. Traditionally, medical education researchers have addressed many critical questions in the effectiveness and efficiency of medical education. What is most lacking are studies exploring in the blueprint and methodology of medical education programs affect the cognitive and clinical competency objectives that are relevant to medical students.

### Principle #4: Design the “Chain of Command” Flow Chart

After identifying the Pillars of the School, each division, center or units must be linked to the accountability organization. A flow chart is often beneficial and maintains the organizational flow of responsibilities even if multiple units or sites are involved in the educating of students. The unification of multiple sites is coordinated with regional leadership reporting to a single departmental leader while all working in unison with a common curricular program, assessments and 3600 evaluation process as directed by the Accountability and Responsibility Policies.

**Figure 1.**
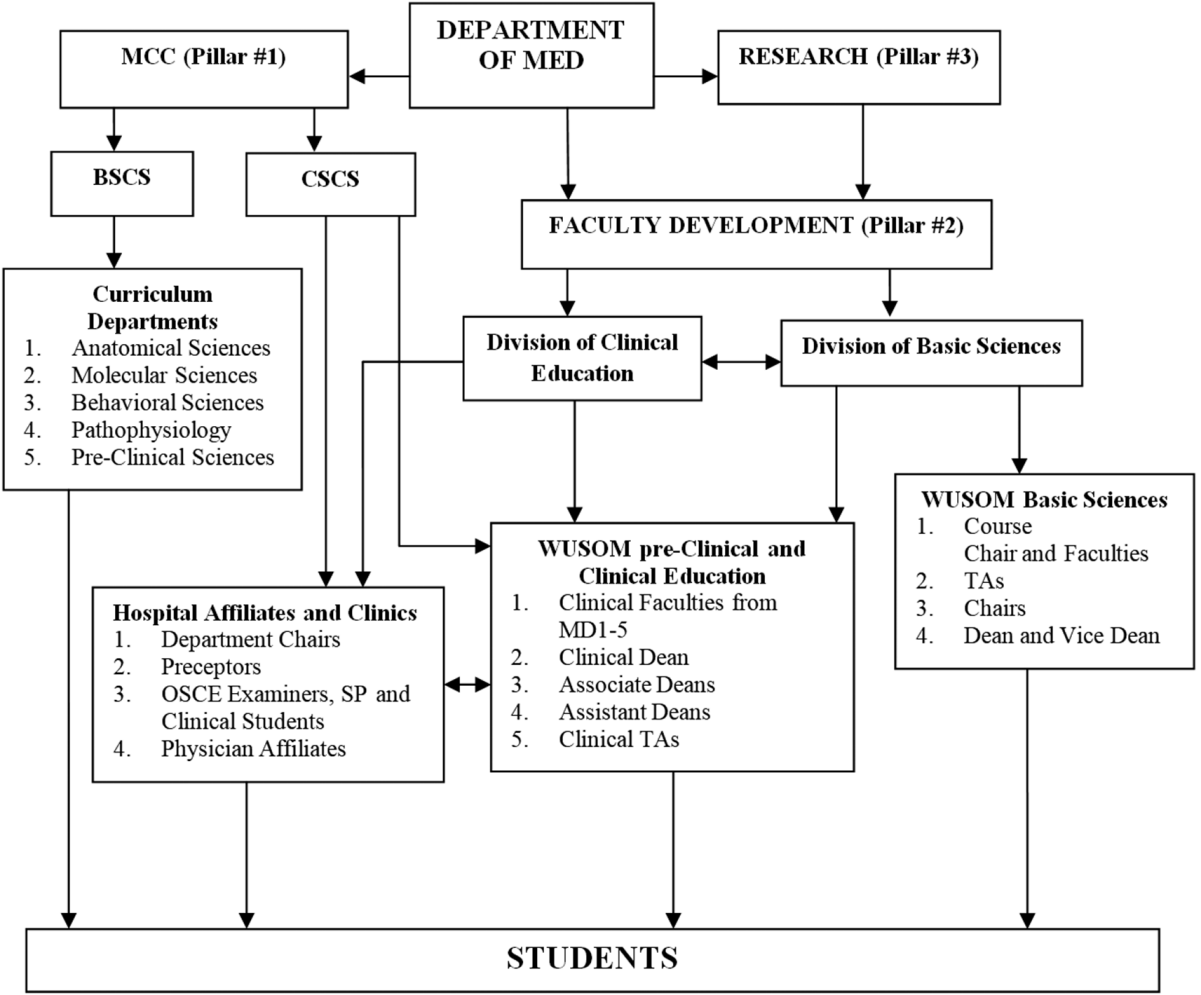
Accountability and responsibility flow chart

### Principle #5: Select a Hierarchy Structure Designation

Every Medical Organization must identify a Hierarchy of Accountability and Responsibility chain that will fit the organizational design based on its Mission, Vision, leadership culture, communication methods, organizational policies, and the ultimate positive and/or negative consequences ( *The Complete Guide to the 5 Types of Organizational Structures for the Future of Work*, 2015). I have provided below a simplistic overview of the five types of hierarchy structures.

**The traditional hierarchy**

The traditional hierarchy is typically identified as a one-way communication flow from the top to the bottom that functions on micro-management without the vision of growth.

**Flatter organizations**

It is an open concept with multiple lines of communication and collaboration between colleagues for a more adaptable, effective, practical, scalable, and logical approach to sharing of ideas, promoting self-worth and recognition within the identifiable team.

**Flat organizations**

It is a self-managed organization corporate structure with no barriers, no job descriptions, managers, or executives. Everyone is equally contributing to the organization.

**Flatarchies**

It is very dynamic in nature and can be thought of as a structure within an organization without a constant limitation or confinement. The Ad-hoc team members can change depending on the demand of the project. The main benefit here is the focus on innovation which is quite a strong competitive advantage in the future of work.

**Holacratic organizations**

Holacracy is a dramatic organization that may require a significant radical change. It is an early and an emerging conceptual structure with a lot of developing ideas.

### Principle #6: Structuring and Empowering of Pillar #1 (EG: MCC)

MCC is probably one of the most important and complex pillar of the entire organization. The Dean is the usual leader carrying out the strategy of the President and the Board of Directors. Like all Medical School, the pre-clinical years 1 and 2 (Basic Science) courses are usually delivered onsite and Clinical years of 3 and 4 (Clinical Clerkship) included various cores, electives and selectives that are either hospital or office based across many states or countries.

The monumental task of membership appointments can render the success or failure of the curricular programs. The MCC must also be charged with accountability and responsibility oversight over all its curricular departments, divisions, sites and individual faculty. The mere development of a curricular program can only be productive when the content, delivery and assessments are all standardized across multiple sites, demonstrating reliability in the delivery of its content according to the learning objectives and the assessment validity are consistent with the material in the course content.

Table 1 below is some of the common suggestions that will help and guide in the development of the MCC Pillar within the Accountable and Responsible Organizational Structure ( *MEDICAL EDUCATION CURRICULUM COMMITTEE*, 2017).

**Table 1.** Accountability charge and authority action

Accountability charge to the Medical Curriculum Committee (MCC)	1. The authority and responsibility of the medical education as a whole; including the overall design, management, integration, evaluation, and enhancement of the Medical Basic Sciences and Clinical Rotation course of studies. 2. Ensures the design and implementation of the medical education departmental objectives, content, review and revise of the curriculum, and establish the basis for evaluating programmatic effectiveness. 3. Uses the internal formative, summative assessments and medical student feedback and external outcome NBME measures to monitor and assess the curriculum, the learning environment, and enhance the quality of the teaching and educational program. 4. Ensures the consistency and standardization of educational experiences while maintain the reliable and valid formative and summative assessments to be rendered at all sites of the school. 5. Ensures the standards of achievement are defined in the student’s hand-book, which is updated according to the governance policies.
Medical Curriculum Committee (MCC) authority actions	1. Defines the learning objectives, integrity, and competencies of the medical education program enables students to achieve those competencies. 2. Oversees, authorizes and/or requires revisions of subcommittee plans for curriculum content and pedagogical methods to be used in each system, course or clerkship. 3. Defines methods for evaluating, assessing, supervising all of its students, faculties, preceptors, and other health professionals. 4. Ensures adequacy of support services and continuous quality improvement of the medical education program by evaluating student performance, teaching effectiveness, and global efficacy to address areas that need improvement. 5. Ensures standardization or comparability of educational opportunities across multiple affiliated sites. 6. Participates in iterative dialogue with the subcommittees, including: a. Review of evaluation data, with discussion of areas that need change as appropriate. b. Identification and communication of opportunities for faculty development and 3600 evaluations. 7. Examine the sub-committee’s internal accountability, strategic plan, and curriculum development while assisting the implementation roll-out and issuing accountability consequences to responsible members. 8. The Committee can establish impromptu or advisory to subcommittees as needed 9. For regular business of the Committee, a quorum shall consist of a simple majority of the membership and a simple majority vote is required for any motion to pass.

### Principle #7: Structuring and Responsibility of subcommittees (BSCS and CSCS)

The Board approves courses and programs recommended directly by the Medical Curriculum Committee through primary advice from subcommittees or mutual agreement with the board and with the assurance that the established policies and procedures have been reviewed and followed. The Board has final approval for educational programs passed by the MCC and MCC assures that the academic and curricular matters will be carried out by the subcommittees in accordance with the final approval. The BSCS is responsible to carry out the entire curricular program for the 1 ^st^ two years and the CSCS is responsible for the 3 ^rd^ and 4 ^th^ years of the Medical Education.

The chair of the subcommittees must have intimate knowledge and be kept informed of the curriculum Standards and assist faculty in the curriculum development process. He leads the organization and supervises the orientation of new members and participates in the on-going training of faculty members as directed by the FDD. In addition, he must have an organized method to perform reviews on technical, prerequisite requirements, multiple sites or distance education programs, general education, library sign-off, articulation, 360 ^0^ Evaluation, overall program success and prepare and sign-off a yearly recommendation report to the MCC.

BSCS:

The subcommittee has been charged with the responsibility for recommending policy and curricular changes to the MCC. These changes must support the institutional mission and vision goals to maximize students’ education and teaching effectiveness. The subcommittee must conduct an annual review of the effectiveness and efficiency of the medical curriculum competency across both years and increase self-focused learning through vertical and horizontal integration. Other responsibilities ( Table 2) include course and examination scheduling for each semester and ensure the course learning objectives are aligned with the school’s educational objectives ( Gorelick, 2019).

**Table 2.** The BSCS curriculum-related activities

1.	Development of the schedule for all courses
2.	Ensuring that the curriculum is logically sequenced
3.	Maximizing horizontal and vertical integration within and across all two years of the curriculum
4.	Evaluation of program effectiveness using outcomes analysis in internal formative and summative assessment and external assessment.
5.	Surveying the content and workload in each course.
6.	Identifying and eliminating gaps and redundancies in the curriculum.
7.	Reviewing the learning objectives of each course and ensuring their alignment with Accreditation Commission of Medical Education critical learning objectives and reaffirm the reliability and validity using mapping of the Program objectives to course contents.
8.	Development of policies that position students for mastery of the school’s educational objectives and maximize their success as learners and future physicians.

The Course Director or the Departmental Head/Chair is the most important link in the Chain of Command. He must maintain Standardization, Reliability and Validity of the Course content, delivery, achieving the goal according to the Learning Objectives and suggesting revisions according to all inputs from everywhere (national benchmark, faculty members and students). He must prepare the 360 ^0^ faculty evaluation report (student evaluations, objective evaluations, and self-evaluation), supervise, carry out consequences, and recommend faculty promotion to the BSCS and MCC.

CSCS:

The primary responsibility of the Clinical Science Curriculum Subcommittee (CSCS) is to direct, review, revise, and supervise the delivery of the clinical curriculum, including the organization of the Pre-Clinical Medical courses during Year 1 and 2. Where possible, foster integration between basic and clinical sciences and assist in the compulsory requirement of electives, selectives and acting internships ( *UGME YEAR 3&4 CURRICULUM SUBCOMMITTEE TERMS OF REFERENCE*, 2019). The Lines of Accountability and Communication of the CSCS; it will take directions from MCC and seeks advice and work collaboratively with the Clinical Department Heads before making recommendations to the MCC. It is always a challenge to have a quorum meeting of the CSCS because of the multiple sites, schedule and responsibility of preceptors, time difference and difficulties with personal commitments. Despite all the above issues, the following suggestions in Table 3 may benefit and improve the quality of the CSCS meetings.

**Table 3.** Clinical Science Curriculum Subcommittee (CSCS) organization

The Clinical Science Curriculum Subcommittee (CSCS) will formally review:	• All Syllabus, course contents, and mode of delivery of the Introduction to Clinical Medicine (ICM) of year 1 and 2, all core-rotations - junior clerkship (year 3) at all campuses and all senior selective and elective courses (year 4) must be evaluated for its standardization, reliability and validity at least once every four years. • Courses or clerkships may be reviewed more than once in four years if recommended by the Subcommittee. • Review reports, including recommendations, will be submitted to the MCC for approval. • All materials used in the formal student/preceptor reviews will be collected and provided to course/clerkship directors, departmental heads, and Associate Dean who will discuss these materials with student representatives and/or preceptors and where possible, and provide an interim report to the Subcommittee, highlighting the impact of concerns. • The Subcommittee will conduct an annual retreat with clerkship directors, departmental representatives, department heads, and others to identify the strengths, weaknesses, opportunities, and threats for the clinical curriculum. • Monitoring and review center of every Clinical Student’s compilation of the Mandatory Clinical Case List/Patient Encounters, MedU, DOCCOM, and log on the commonly observed and/or performed Clinical Procedures during every core-rotation. • The preceptor’s satisfactory signature is required before the student may advance to the next core-rotation. • Monitor the students’ attendance accountability to clinical-rotation, Webinars, supplemental clinical practice, and preceptors’ didactic lectures • Core preceptors are responsible in the direct observation of history taking and physical examination accustom to each core-rotations. • Mid-rotation evaluation is required for rotations longer than four weeks. • Formative and Summative Clinical assessments are delivered timely and so that students can receive final grades within Two (2) weeks of completion of the rotation.
The Clinical Science Curriculum Subcommittee (CSCS) meeting suggestions:	• Meet during Associate Dean’s site visit (per semester or every 3 months) • Meet during OSCE final exam (Clinical Department Heads are the examiners for the Final OSCE). • A quorum and approval will consist of a simple majority of the voting members. • The CSCS can appoint task forces to investigate particular issues, and, on the basis of information provided by a task force, recommendations to the Senior Associate Dean or the Dean. • The CSCS can appoint non-voting, ad-hoc members when their expertise is needed. Proposals will be considered and recommendations to approve or not approve will be voted on. • Decisions reached by the CSCS will be forwarded directly to the MCC.

### Principle #8: Relationship between Subcommittees and Departments

The Clinical Dean and Associate Dean of Clinical Education in the CSCS working in accordance with the Associate Dean of Clinical Academic Affairs to strategize an effective Accountable and Responsible Plan that will be implemented across different sites for a comparable Clinical Curriculum Program for the 3 ^rd^ & 4 ^th^ Year. The Associate Dean of Clinical Education must maintain an open communication receiving and directing suggestions from Departmental and Clinical Site Directors for changes to the Clinical Curriculum, ensuring Years 3 & 4 learning objectives (syllabus) are delivered reliably across different sites and that the validity of the Clinical Assessment Objectives are psychometrically analyzed and mapped in accordance with the Accreditation Commission of Medical Education. Any changes in assessments if required are approved by the CSCS before forwarding to the MCC for the final approval ( *UGME YEAR 3&4 CURRICULUM SUBCOMMITTEE TERMS OF REFERENCE*, 2019).

### Principle #9: Implementation of Accountability within the BSCS and CSCS departments

BSCS:

An Accountability and Responsibility Plan is not a plan without an immediate implementation with supporting consequences. A strategic plan outlining methods of supervision and 360 ^0^ evaluation of every departmental member’s accountability and responsibility and subsequent consequences must be immediately agreed and acknowledged between the Department Chair and its Departmental Faculties.

It is imperative that the Course Director will develop a purposefully required frequency of formative assessment methods, effective communication channels and a “watch list” to identify weak students at or before the block I exam and throughout the entire semester. Within 5 days after the identification of a weak student, these students must be integrated into methods of coaching which can be student tutoring program, one-on-one review, study specialist, and/or small group assistance prior to remediation exams.

Senior faculty must supervise and assess the junior faculty’s in-classroom pedagogy effectiveness, the percentage of student-focused and topic directed Problem Based Learning (PBL), the frequency of formative and summative assessments using a bank of questions that are reliable and valid in line with the course learning objectives as directed by the Medical Education learning criteria.

CSCS:

When Residents, Fellows and non-Clinicians are involved in the clinical education of the students, the Departmental Heads or Preceptors must ensure that these appointees are orientated to the students’ learning objectives, the Mandatory Clinical Case List/Patient Encounters, and Clinical Procedures as mandated in the syllabus for each core-rotation and/or selective choices. CSCS must review and approve new electives offering and periodically reviews the existing inventory of the University’s electives for ongoing quality assurance and making sure an effective system for elective, selective and career advising is in place.

In accordance to the Student Advising and Support Program, CSCS and Department Head working with the hospital will promote a respectful learning environment, identifies and promptly addresses student mistreatment, student/preceptor conflict, and other violations of the Faculty’s Professional Standards. CSCS is also responsible with the contractual agreements for the education sites, Preceptors, Site coordinators and Departmental Heads. The yearly contractual agreements are reviewed and renewed based on the student evaluation reports during the core rotation of their educational experiences in Years 3 & 4.

Any revision and a new proposal to the Clinical Education Program for the Years 3 & 4 curricula, the CSCS must make recommendations for approval to the MCC. CSCS with the assistance of the Clinical Department Heads will devise a plan of implementation, informs FDD of current and emerging requirements to prepare Preceptors to deliver curriculum and assessments within Years 3 and 4. Post-implementation analysis report on the success/failure of the changes with a recommendation from pilot to operations will be submitted to the MCC. Any recommended changes for the Clinical Years curriculum components must comply with the relevant Accreditation Committee, Higher Education Accreditation standards and University policies.

### Principle #10: Structuring and Empowering of Pillar #2: Faculty Development Program (FDP)

There has been a shift and development of integrating medical curricula with newer methods of curriculum pedagogy and appropriate assessment methods. Faculty Development Department are expected to train their faculty members to expand new skills that are vitally important to these changes.

Faculty development is a progression to self-improvement for medical faculties and preceptors to advance their skills in the educational leadership, scholarly activities, personal development, and professional academic skills ( Damp et al., 2016). Faculty development activities are successful when individual goals of teaching, instructional curriculum design, scholarly activities, leadership and personal and professional development are met ( *Office of Faculty Development/Home | College of Medicine*, 2019).

The FDP must cultivate an educational environment in which faculties and preceptors feel empowered to strive for excellence and work toward the educational culture of the school. The FDP’s mission should relate directly to the ultimate goal of effective education at all levels of training in the continuum of medical education. This will maximize every student’s potential by achieving Competency Objectives set in the Medical Education Goals of the Institution.

A. **How to establish FDP:** FDP vary in structure and function between institutions, there is no one ideal model and all programs have advantages and disadvantages. Any variable preferences will depend on specific institutional factors including but not limited to financial, administrative support, human resources, campus resources, and expertise of the faculty or staff members ( Kamel, 2016).

FDP could be established through the activities that supported the academic mission, vision, and values of the institution. Programs are designed to improve teaching and learning ranged from a one-time activity to regularly scheduled workshops or seminars catering to institutional members or openly competitive scholar programs with supervised learning and assessments (Clinical Teaching Program - Stanford Faculty Development Center for Medical Teachers - Stanford University School of Medicine, 2019; Office of Faculty Development/Home | College of Medicine, 2019). A supervised training program for the probation faculty members should be effectively implemented to achieve a successful outcome in the form of increased interest for teaching, research and publication while focusing on the institutional goals of standardization, reliability and validity. ( Table 4)

**Table 4.** Types of Faculty Development Programs/Services

1.	Workshops and Seminars (examples below) • Writing meaningful learning objectives • Writing test items to measure learning objectives • Making your presentations interactive • Designing effective instruction • The Myers Briggs type indicator applied to teaching • 7 Habits applied to teaching • Developing a teaching portfolio • Computing / Medical Informatics skills
2.	Book discussion groups
3.	360 ^0^ Observation and Feedback
4.	Individual consultations
5.	Peer coaching
6.	CD’s on clinical teaching
7.	Monthly educational publications
8.	Web Based (Online) faculty development materials
9.	Notification to faculty of faculty development programs offered by main campus in the following categories: • Academic course portfolios • Basic teaching and learning in medicine • Problem Based Learning (PBL) • Large and small group teaching • Scholarship of teaching and learning
10.	Principles on which the faculty development program is based on: • Strong administrative support • Reward structures for participation in faculty development programs • Teaching viewed as a scholarly activity • Systematic skills development • Based on principles of adult learning • Sensitive to identified needs • Participants learn from each other • Atmosphere of caring and trust • Based on collaboration, teamwork, and shared vision • Celebration of successes

B. **Structure of FDP:** The FDP’s conceptual intentional approach to faculty education using short and focused series of training workshops that can provide the opportunity to practice sound teaching principles, educational techniques and specific topics in newly acquired skills. Several research studies demonstrated that such programs had variety of successes improving attitudes, self-effectiveness, and teaching behavior facilitating faculty’s ability to recognize and increasing knowledge of teaching philosophy ( Bigby and Barnes, 1993; O’Connor, Bigby and Gallagher, 1993; Kamel, 2016).

These short-term programs provide very limited content and teaching objectives, such as clinical skills, curriculum design, and effective assessments ( Steinert et al., 2006; McLeod et al., 2008). The selection of which program is most suitable regarding workshops and seminar programs is that they should be analyzed and planned in response to the needs of institutional goal and faculty member specific to the student success ( Bergquist and Phillips, 1975; Ullian and Stritter, 1997; Wilkerson and Irby, 1998).

### Principle #11: Structuring and Empowering of Pillar #3 (eg. Research)

A. **Research Methodology:**

Research Projects are so diverse. The researcher has to consider and identify the methodology that is appropriate for his/her selected project. The researcher must be clear about the purpose of the research and formulating a research hypothesis. In order to ensure that the research project is in compliance with the University and its external agency regulations, the University must establish a chain of command for oversight of all such projects ( *Research planning*, 2019; *Roles and responsibilities in research administration*, 2019).

**Table 5.** Research methodologies and responsibilities

Examples of methodologies include:	• Case series/case note review • Cohort observation • Controlled trial without randomization • Epidemiology • Qualitative research • Observational research • Randomized controlled trial • Interview • Questionnaires • A mix of methodologies may be used
Chain of Command responsibility:	• Senior university administrators • Pre award management • Post award management • Deans • Department Chairs • Principal Investigators (PI) • Project Staff • Sponsor • Sponsor’s legal representative • Chief investigator • Principal investigator • Data controller

Relevant supervisors have the responsibility to encourage, develop and lead research projects that individual students at the graduate level and below can effectively contribute at different stages. Undergraduate students should only conduct research projects under supervision that will directly involve patient contact, public health issues, or social service settings to mitigate any legal risks. An organized research culture will encourage interest and participation amongst relevant undergraduate students by enabling them to develop skills leading to ingenuous research way. Students from select research topic that is primarily related to non-medical healthcare issues should have a co-supervisor with relevant experience that will guide them to understand the context and the associated research process.

### Principle #12: Development and approach to accountability and responsibility consequences

MED must ensure that all pillars of the departments provide appropriate supervision for all faculties and staffs in a work environment that is consistent with proper leadership, the educational needs of the medical students, and the applicable accreditation requirements. It is the expectation of any university where all faculties will adhere to the university guidelines and policies according to the accountability and responsibility policies. The appointed chain of command has both an ethical and a legal responsibility for the overall education of the medical students and for the supervision of the subordinates involved in the success of the university.

In the Medical Education learning environment, every student during the basic science course and the clinical clerkship must have an identifiable, appropriately-credentialed and qualified faculty or preceptor who is ultimately responsible for that course or core-rotation experience. Preceptor and faculty members must be informed of their respective roles in that student’s medical education when providing direct education to the student. Clinical Department Chair must demonstrate that the appropriate level of supervision is in place for all faculty and Preceptors who educate the assigned course or core-rotation.

The Curricular Subcommittees (BSCS and CSCS) will supervise the accountability and responsibility policies. A chain of command that emphasizes authority and responsibility as judged on the subordinates must be made by the Chair of the Subcommittees who is ultimately responsible for the overall education of the student. Such judgments shall be based on the Department Chair, Associate Dean of Clinical Education/Academic Affairs, student’s evaluation and 360 ^0^ evaluations from other affiliates providing direct observation and assessments of each preceptor’s skills, ability, knowledge, accountability and responsibility. The Chain of Command must demonstrate that the appropriate level of supervision is in place for all members of the subcommittees. Supervision may be exercised through a variety of methods, as appropriate to the situation.

The level of responsibility accorded to each faculty must be supervised by the Department Chair. The Department Chair will conduct a fair accountability assessment of its faculty members using sufficient direct supervision and gathering of 360 ^0^ evaluation-assessments assisting in the preparation of the faculty promotion report to the MCC. This same report constitutes the decision for promotional recommendation by the Dean.

## Conclusion

Almost every school has organizational Hierarchy appointments, handbooks, policies and a specific culture of Human Resources. The complexity of its institutional directives is exponentially co-related to the age of its foundation, number of student enrolments and layers of organizational leadership. The accountability and responsibility of its faculties and staff diminish with each additional layer of leadership and diluting the personal accountability with every addendum to the institutional policies. In this paper, the simplicity of the core pillars and its branches are explored and explained. The Chain of Commands is linked with specific responsibility charges to ensure subordinate competency, motivating self-development and growth for a common goal of students’ higher education.

One of the biggest accreditation issues with multiple medical training sites has been identified as the lack of comparable unity of education, unreliable teaching methods and inconsistent validity of its assessment to the content of its learning objectives. Sharing my personal experience and others, I have shared 12 simple principles on the designing of Accountability and Responsibility plan that will cross distance and linking the chain of command despite distance and multiple education sites. The ultimate success of the Chain of Command requires the unwavering support of the upper hierarchy, total acceptance of its members and the unmatched selfless efforts of its Departmental Chairs.

## Take Home Messages

• This paper is intended for New, Joint and International medical school operating from multiple sites.
• “One-University” must operate with a clear and visible flow of Accountability and Responsibility between multiple sites under one seamless organization.
• Our paper provides the basic foundation and clarifies the methodology to meet the “One-University” standard.

## Notes On Contributors

Dr. Lee HangFu is the Associate Dean of Clinical Academic Affair with the Windsor University School of Medicine. His major accomplishment is the ongoing concept of the “One University” cohesiveness. He has amalgamated the school Clinical Curriculum Program (inclusive of PBL, TBL, and SBL), Policies, Learning Objectives, OSCE Advancement and Assessments, and Psychometric Analysis between Chicago, Houston, Jamaica and St. Kitts. He has collaborated with the Preceptors, Clinicians and Departmental Chairs through Faculty Development for a comparable clinical education in standardization, reliability and validity as required by the Accreditation Committee. ORCID: https://orcid.org/0000-0001-7626-6268.

Dr. Samal Nauhria M.D. Pathology is Chair of Pathophysiology and his current research is based on the use of technology in medical education.
