# Supplementary material for: Principles for designing accountability and responsibility plan in medical education that spreads across multiple sites
Source: MedEdPublish (2016). 2019 Mar 15;8:57. [Version 1] doi: 10.15694/mep.2019.000057.1 (PMC10712624; doi:10.15694/mep.2019.000057.1)
Supplement: Peer review history

# Reviewer report: Reviewer response for version 1

**DOI:** 10.21956/mep.19781.r29729

**Published:** 2019-03-21

**Authors:** Felix Silwimba

**Affiliations:** 1 University of Lusaka

This review has been migrated. The reviewer awarded 5 stars out of 5 this is a well explained report on organizing medical education and collaboration within the medical school. I have learnt a lot and I have been reminded of what to do right.

Reviewer Expertise:

NA

No decision status is available

# Reviewer report: Reviewer response for version 1

**DOI:** 10.21956/mep.19781.r29730

**Published:** 2019-03-16

**Authors:** Sateesh Babu Arja

**Affiliations:** 1 Avalon University School of Medicine

This review has been migrated. The reviewer awarded 5 stars out of 5 The title of the article designing accountability and responsibility of medical education across multiple clinical sites got my attention. It is very well written paper. Even though the article is titled for multiple clinical sites, this paper covered the principles of entire medical program including basic sciences specially for offshore Caribbean medical schools. The tables provided are very informative. The figure 1 mentioned in this paper might be the organizational chart followed at Windsor Univesity. It is interesting to see that Medical Education Department oversights the curriculum committee, faculty development activities, and even the research activities. Uniform and standardized curriculum, learning objectives, assessments, and core clinical encounters or requirement of log boos/ work diaries can help in establishing the uniform clinical experiences across multiple clinical sites.This paper is useful for all academic leaders especially for offshore Caribbean medical schools.

Reviewer Expertise:

NA

No decision status is available

# Reviewer report: Reviewer response for version 1

**DOI:** 10.21956/mep.19781.r29731

**Published:** 2019-03-15

**Authors:** P Ravi Shankar

**Affiliations:** 1 American International Medical University

This review has been migrated. The reviewer awarded 4 stars out of 5 Thank you for the opportunity to review this interesting manuscript. The authors try to address the major challenge of ensuring comparable educational experiences among multiple learning sites. Offshore Caribbean medical schools (OCMS) face major challenges in this regard. The authors put forward twelve principles to assist educators during this task. The manuscript is well-written. One possible problem is that there may be multiple ways to attain a particular objective and different organizational systems which may work for different institutions. For example consider the basic science and clinical science subcommittees of the curriculum committee. Some schools may not have subcommittees and the curriculum committee is responsible for oversight of the entire program. Many offshore schools have small faculty bodies and hence the curriculum committee may not be large enough to have many subcommittees. The organization structure of schools may also be different. Accrediting bodies usually recommend the creation of a Board of Governors who does not have financial interests in the school to guide the school operations. I have not come across the term Accreditation Commission of Medical Education before. Is it a standard term? The tables are informative. Faculty members and the educational leadership of schools operating at multiple sites will find this manuscript to be of interest.

Reviewer Expertise:

NA

No decision status is available
